# Supplementary material for: Excess of genomic defects in a woolly mammoth on Wrangel island
Source: PLoS Genet. 2017 Mar 2;13(3):e1006601. doi: 10.1371/journal.pgen.1006601 (PMC5333797; doi:10.1371/journal.pgen.1006601)
Supplement: S1 Text — (PDF) [file pgen.1006601.s001.pdf]

Supporting Information

Analysis of samples M4 and M25

We aligned all major runs in the SRA for two *M. primigenius* specimens previously published, M4 and M25 (Table S8) (Lynch et al. 2015). As a comparison for sequence quality, we also aligned and analyzed reads for one female *E. maximus indicus* specimen, Maya, sequenced and processed in the same study. Previously published sequences for all three elephantids were aligned to the *L. africana* r.4.0 reference genome using bwa 0.7.12-r1044 (Li and Durbin 2009), with parameters set according to (Palkopoulou et al. 2015) `bwa aln -l 16500 -o 2 -n 0.01`. Indels were identified and realigned using GATK as defined above. We then generated all SNPs using samtools mpileup (-C50 -u -g) and consensus fastq was generated using bcftools consensus caller (bcftools call -c) and bcftools vcf2fq.pl with a minimum depth threshold of 3 reads and a maximum depth of twice the mean coverage for each genome. Resulting fastq files were converted to psmcfa using the PSMC toolkit (Li and Durbin 2011). We then ran PSMC (Li and Durbin 2011) exactly as described in Palkopoulou et al. (2015), with 64 time intervals, `(-p "4+25*2+4+6")`.

Demographic inference for mammoth samples from Oimyakon and Wrangel Island (Palkopoulou et al. 2015) show $N_e \leq 25,000$ (Figure S4). Analysis of samples M25 and M4 suggests N_e in the range of 10^{10}-10^{11} over the history of woolly mammoths (Figure S4), a result that is inconsistent with estimates based on mtDNA (Barnes et al. 2007) or habitat availability (Nogués-Bravo et al. 2008). Demographic inference for Maya the elephant yields $N_e < 20,000$, with a bottleneck event roughly 200,000 years ago.

Given the inconsistencies in the M4 and M25 results, we examined heterozygosity data more directly for each of the samples, using chromosome 1 as an example dataset. We calculated heterozygosity for 10 kb windows in each mammoth and elephant sample. M4 and M25 both display high heterozygosity. We observe 30 heterozygous sites per 10 kb window in M4, and 38 heterozygous sites per 10 kb window in M25. These numbers are 2-3 fold higher than the observed mean of 11-14 sites per 10 kb window in Wrangel, Oimyakon, and Maya (Table S9; Figure S5). The abnormally high heterozygosity is likely to explain abnormal estimates of N_e from PSMC. We then examined support for heterozygous SNP calls, using the first 5000 SNPs on chromosome 1 as a test set. If sites are truly heterozygous, there should be symmetrical support for each base by site. We identified sites with significantly skewed support in a binomial test. Mammoth specimens M4 and M25 from (Lynch et al. 2015) have an excess of SNPs with significantly asymmetrical support compared to the Oimyakon and Wrangel mammoths, as well as Maya the elephant (Table S10; Figure S6A-S6E). There is a greater number of asymmetric sites that favor the reference allele than the non-reference allele in both M4 and M25 (Table S10; Figure S6A-S6B). Such asymmetry would be expected if some other elephantid DNA had contaminated these two samples, or if in vitro recombination occurred between barcodes during PCR amplification or sequencing. Removing A/G and T/C mutations did not correct the pattern, suggesting that these results are not a product of differences in damage for archaic samples (Figure S7). Multiple mammoths were sequenced in the lab, only some of which have been published (http://mammoth.psu.edu/moreThanOne.html; accessed June 18, 2016). We are currently

unable to examine all potential sources of contamination. These results left us concerned for the quality of the sequences. Hence, we did not include the two mammoth specimens M4 and M25 in the current genomic analysis of deletions, retrogenes, stop codons, or amino acid substitutions.
