# Supplementary material for: Excess of genomic defects in a woolly mammoth on Wrangel island
Source: PLoS Genet. 2017 Mar 2;13(3):e1006601. doi: 10.1371/journal.pgen.1006601 (PMC5333797; doi:10.1371/journal.pgen.1006601)
Supplement: S1 Table — (PDF) [file pgen.1006601.s002.pdf]

Table S1: Non-synonymous and synonymous sites

		Wrangel	Oimyakon[1]
Heterozygous	Non-synonymous	12784	9445
	Synonymous	10231	8913
Homozygous	Non-synonymous	16149	13447
	Synonymous	21842	18950

[1] Raw numbers, without correction for changes in coverage.
