# Supplementary material for: Excess of genomic defects in a woolly mammoth on Wrangel island
Source: PLoS Genet. 2017 Mar 2;13(3):e1006601. doi: 10.1371/journal.pgen.1006601 (PMC5333797; doi:10.1371/journal.pgen.1006601)
Supplement: S2 Table — (PDF) [file pgen.1006601.s003.pdf]

Table S2: DAVID Gene ontology for premature stop codons in the Wrangel Island Mammoth

Specimen	Function	EASE score
Oimyakon	Olfactory receptors	4.1
Wrangel	Olfactory receptors	9.1
	Ankyrin domains	1.6
