# Supplementary material for: Excess of genomic defects in a woolly mammoth on Wrangel island
Source: PLoS Genet. 2017 Mar 2;13(3):e1006601. doi: 10.1371/journal.pgen.1006601 (PMC5333797; doi:10.1371/journal.pgen.1006601)
Supplement: S3 Table — (PDF) [file pgen.1006601.s004.pdf]

Table S3: Heterozygosity for Deletions Identified in Elephantid Genomes

		Oimyakon	Wrangel	Maya
All	Homozygous[1]	5035	6147	5141
	Hemizygous	16223	21081	22954
Exon Deletions	Homozygous	136	173	165
	Hemizygous	1347	1985	4248

[1] $\leq 10\%$ coverage
