# Supplementary material for: Excess of genomic defects in a woolly mammoth on Wrangel island
Source: PLoS Genet. 2017 Mar 2;13(3):e1006601. doi: 10.1371/journal.pgen.1006601 (PMC5333797; doi:10.1371/journal.pgen.1006601)
Supplement: S4 Table — (PDF) [file pgen.1006601.s005.pdf]

Table S4: Shared Deletions Identified in Mammoth Genomes

	Mutations
Homozygous[1]	3001
Heterozygous	9581
Mixed	877
Total	13459

[1] $\leq 10\%$ coverage
