# Supplementary material for: Excess of genomic defects in a woolly mammoth on Wrangel island
Source: PLoS Genet. 2017 Mar 2;13(3):e1006601. doi: 10.1371/journal.pgen.1006601 (PMC5333797; doi:10.1371/journal.pgen.1006601)
Supplement: S5 Table — (PDF) [file pgen.1006601.s006.pdf]

Table S5: DAVID Gene ontology for deleted exons

Specimen	Function	EASE score
Oimyakon	Cell junction	4.6
	Neurons	3.42
	Zinc fingers	3.41
	Aldo/keto metabolism	3.10
	Calcium dependent transport	2.91
	DNA damage	2.85
	Transcription regulation	2.71
	Development	2.66
Wrangel	Major Urinary proteins	7.95
	Pleckstrins	5.49
	Transcription regulation	4.86
	Cellular transport	3.51
	DNA damage	3.34
	Chromatin regulation	3.15
	Hox domains	3.06
	Development	2.75
