# Supplementary material for: Excess of genomic defects in a woolly mammoth on Wrangel island
Source: PLoS Genet. 2017 Mar 2;13(3):e1006601. doi: 10.1371/journal.pgen.1006601 (PMC5333797; doi:10.1371/journal.pgen.1006601)
Supplement: S6 Table — (PDF) [file pgen.1006601.s007.pdf]

s

Table S6: DAVID Gene ontology for retrogenes in the Oimyakon Mammoth

Function	EASE score
Ribosome	6.3
Post translational modification	4.4
Lipoproteins	3.4
Spliceosome	3.1
RNA binding	2.6
Lipoprotein metabolism	2.2
Nucleolus	2.0
Glutamine metabolism	1.9
Aspartate metabolism	1.8
Starch and drug metabolism	1.7
Proteasome	1.6
Translation initiation	1.6
