# Supplementary material for: Excess of genomic defects in a woolly mammoth on Wrangel island
Source: PLoS Genet. 2017 Mar 2;13(3):e1006601. doi: 10.1371/journal.pgen.1006601 (PMC5333797; doi:10.1371/journal.pgen.1006601)
Supplement: S7 Table — (PDF) [file pgen.1006601.s008.pdf]

Table S7: DAVID Gene ontology for retrogenes in the Wrangel Island Mammoth

Function	EASE score
Ribosome	8.3
Ubl conjugation	6.8
Spliceosome	4.3
Translation initiation	2.8
Lipoprotein	2.6
Nuclear body	2.3
Cytoskeleton	2.0
Aminoacylation	1.8
HEAT elongation	1.6
RNA splicing	1.6
