# Supplementary material for: Excess of genomic defects in a woolly mammoth on Wrangel island
Source: PLoS Genet. 2017 Mar 2;13(3):e1006601. doi: 10.1371/journal.pgen.1006601 (PMC5333797; doi:10.1371/journal.pgen.1006601)
Supplement: S8 Table — (PDF) [file pgen.1006601.s009.pdf]

Table S8: SRA and ENA Identifiers for Mammoth and Elephant Sequence Data

Specimen	Database ID
Oimyakon	ERR852028
Wrangel	ERR855944
Maya	SRX1015606
	SRX1015608
M4	SRX1015711
	SRX1015712
	SRX1015714
	SRX1015715
	SRX1015717
	SRX1015679
	SRX1015671
	SRX1015640
	SRX1015634
	SRX1015625
M25	SRX1015733
	SRX1015732
	SRX1015729
	SRX1015727
	SRX1015726
