# Supplementary material for: Excess of genomic defects in a woolly mammoth on Wrangel island
Source: PLoS Genet. 2017 Mar 2;13(3):e1006601. doi: 10.1371/journal.pgen.1006601 (PMC5333797; doi:10.1371/journal.pgen.1006601)
Supplement: S9 Table — (PDF) [file pgen.1006601.s010.pdf]

Table S9: Heterozygous sites per 10 kb

Hets	Specimen
12	Wrangel
14	Oimyakon
11	Maya
30	M4
38	M25
