# Supplementary material for: Excess of genomic defects in a woolly mammoth on Wrangel island
Source: PLoS Genet. 2017 Mar 2;13(3):e1006601. doi: 10.1371/journal.pgen.1006601 (PMC5333797; doi:10.1371/journal.pgen.1006601)
Supplement: S10 Table — (PDF) [file pgen.1006601.s011.pdf]

Table S10: Asymmetrical Support

Asymm SNPs	Favor Ref	Favor Alt	Specimen
498	166	332	Wrangel
217	59	158	Oimyakon
377	240	137	Maya
1355	1179	176	M4
2383	1859	524	M25
