# Supplementary material for: Excess of genomic defects in a woolly mammoth on Wrangel island
Source: PLoS Genet. 2017 Mar 2;13(3):e1006601. doi: 10.1371/journal.pgen.1006601 (PMC5333797; doi:10.1371/journal.pgen.1006601)
Supplement: S1 Fig — (PDF) [file pgen.1006601.s012.pdf]

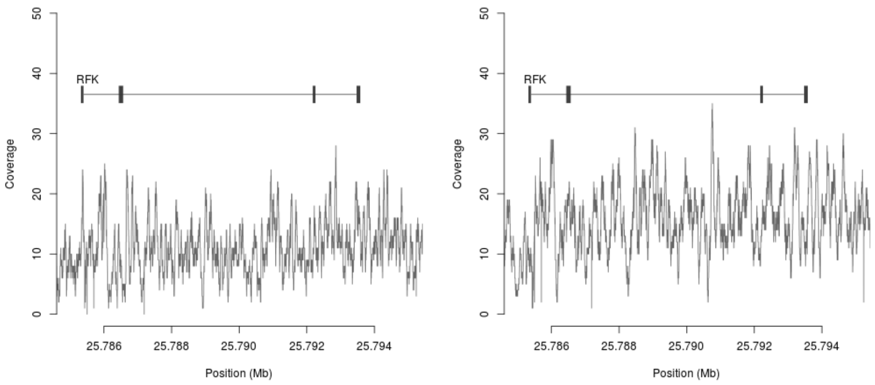

(A) Oimyakon (B) Wrangel Island

Figure S1: Coverage depth at the *RFK* locus in the A) Oimyakon mammoth and B) Wrangel Island Mammoth. There is a 50% reduction in coverage at the first exon of *RFK* in the Wrangel Island mammoth but not in the Oimyakon mammoth.
