# Supplementary material for: Excess of genomic defects in a woolly mammoth on Wrangel island
Source: PLoS Genet. 2017 Mar 2;13(3):e1006601. doi: 10.1371/journal.pgen.1006601 (PMC5333797; doi:10.1371/journal.pgen.1006601)
Supplement: S2 Fig — (PDF) [file pgen.1006601.s013.pdf]

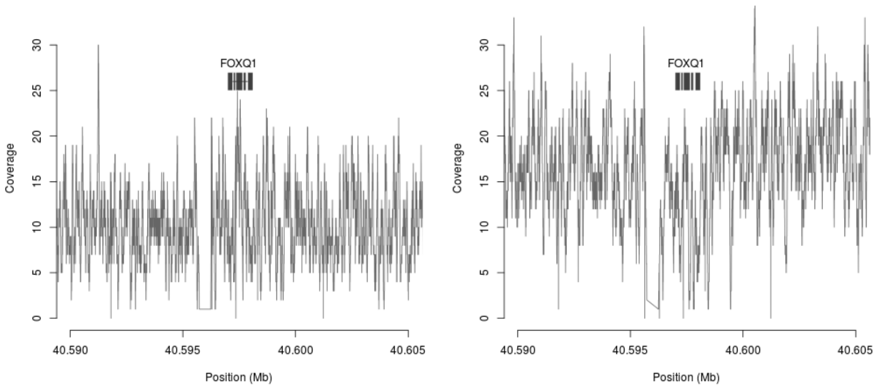

(A) Oimyakon (B) Wrangel Island

Figure S2: Coverage depth at the *FOXQ1* locus in the A) Oimyakon mammoth and B) Wrangel Island Mammoth. There is a 50% reduction in coverage at *FOXQ1* in the Wrangel Island mammoth but not in the Oimyakon mammoth.
