# Supplementary material for: Excess of genomic defects in a woolly mammoth on Wrangel island
Source: PLoS Genet. 2017 Mar 2;13(3):e1006601. doi: 10.1371/journal.pgen.1006601 (PMC5333797; doi:10.1371/journal.pgen.1006601)
Supplement: S3 Fig — (PDF) [file pgen.1006601.s014.pdf]

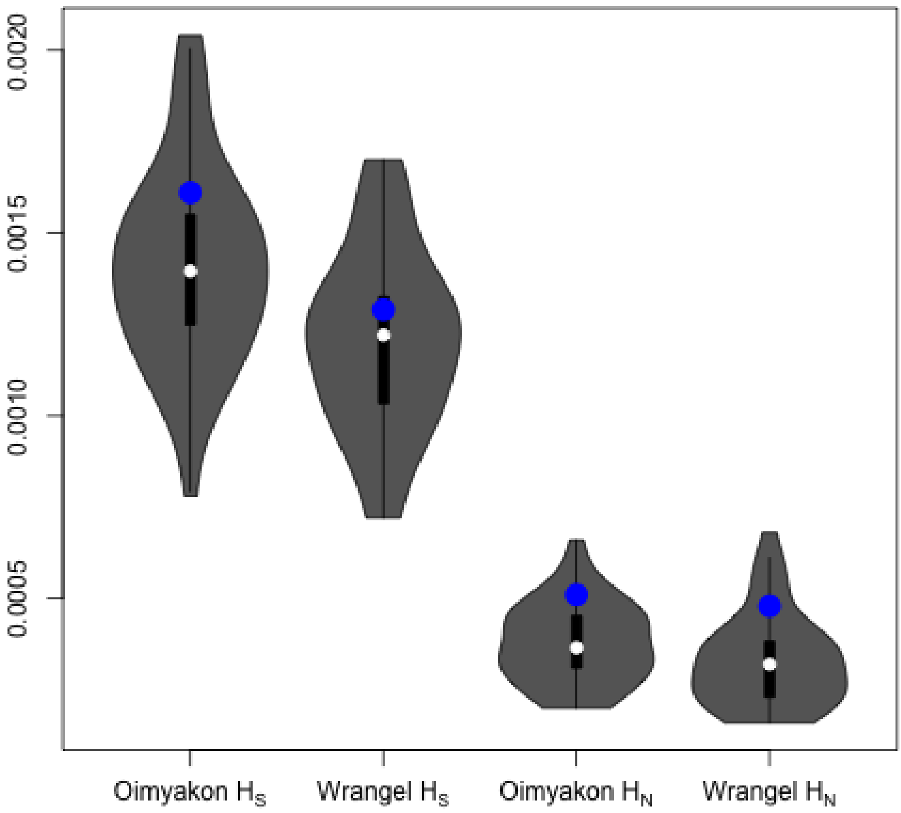

Figure S3: Simulations for heterozygosity at synonymous and non-synonymous sites for the Oimyakon and Wrangel Island mammoths. Black bars show upper and lower quartiles. The white dot is the median. Grey fields show the full distribution of datapoints. Empirical values for the genome wide average are shown in blue.
