# Supplementary material for: Excess of genomic defects in a woolly mammoth on Wrangel island
Source: PLoS Genet. 2017 Mar 2;13(3):e1006601. doi: 10.1371/journal.pgen.1006601 (PMC5333797; doi:10.1371/journal.pgen.1006601)
Supplement: S4 Fig — (PDF) [file pgen.1006601.s015.pdf]

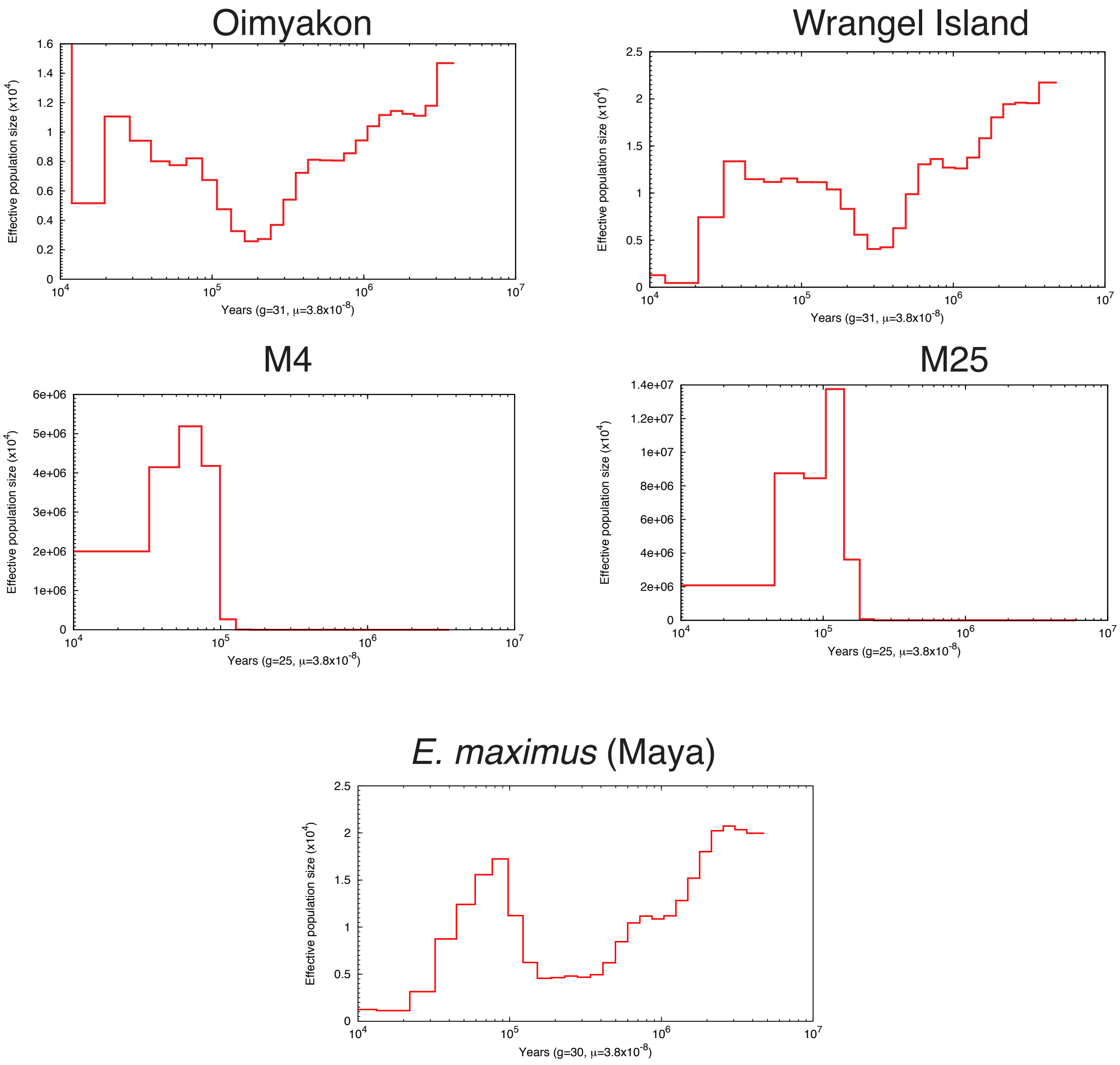

Figure S4: PSMC results for four woolly mammoths and one elephant. M4 and M25 both display effective population sizes of 10^{10} or higher.
