# Supplementary figures and images for: Excess of genomic defects in a woolly mammoth on Wrangel island
Source: PLoS Genet. 2017 Mar 2;13(3):e1006601. doi: 10.1371/journal.pgen.1006601 (PMC5333797; doi:10.1371/journal.pgen.1006601)

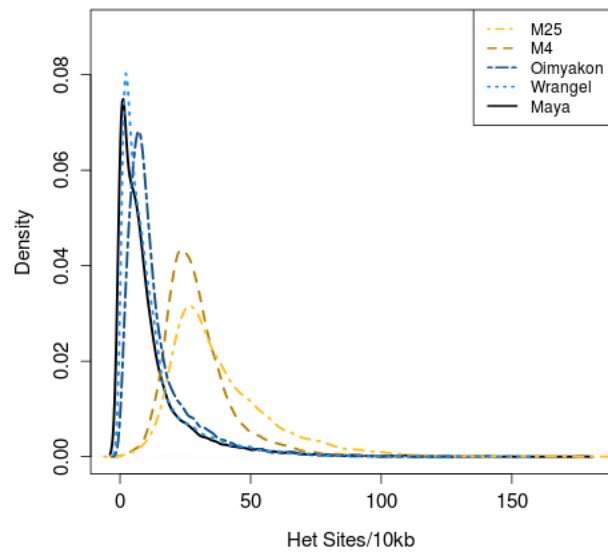

Figure S5: Heterozygosity for mammoth and elephant samples.

Supplement: S5 Fig — (PDF) [file pgen.1006601.s016.pdf]
