# Supplementary material for: Excess of genomic defects in a woolly mammoth on Wrangel island
Source: PLoS Genet. 2017 Mar 2;13(3):e1006601. doi: 10.1371/journal.pgen.1006601 (PMC5333797; doi:10.1371/journal.pgen.1006601)
Supplement: S6 Fig — (PDF) [file pgen.1006601.s017.pdf]

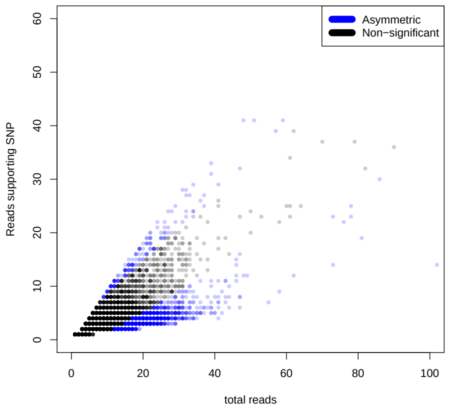

(A) M4

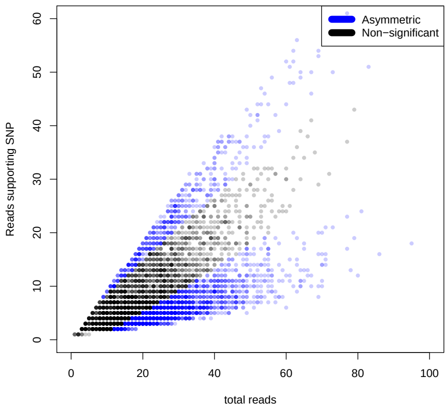

(B) M25

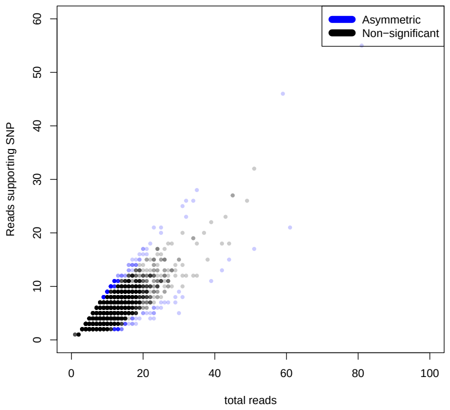

(C) Oimyakon

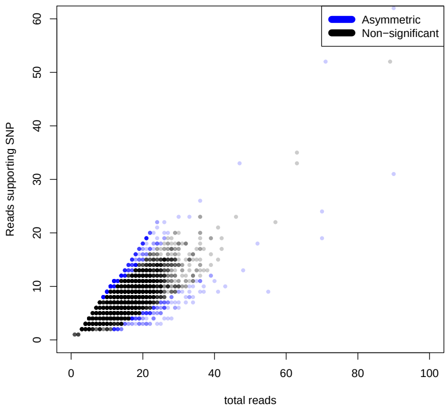

(D) Wrangel Wrangel

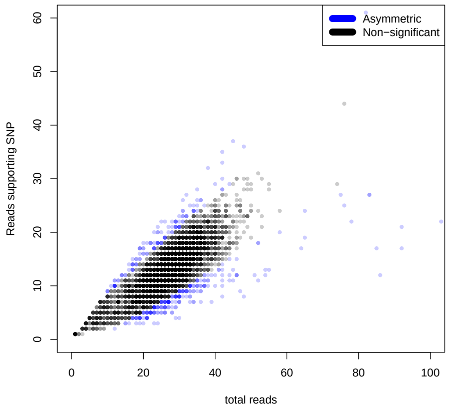

(E) Maya

Figure S6: Asymmetric SNPs out of 5000 representative SNPs on chromosome 1.
