# Supplementary material for: Excess of genomic defects in a woolly mammoth on Wrangel island
Source: PLoS Genet. 2017 Mar 2;13(3):e1006601. doi: 10.1371/journal.pgen.1006601 (PMC5333797; doi:10.1371/journal.pgen.1006601)
Supplement: S7 Fig — (PDF) [file pgen.1006601.s018.pdf]

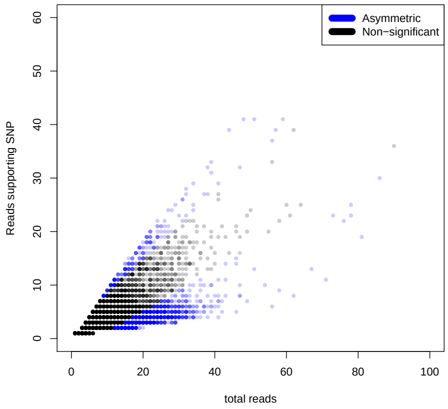

(A) M4

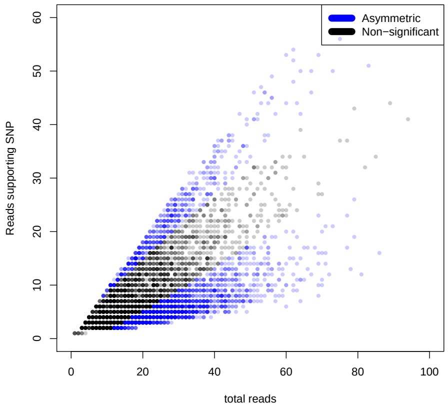

(B) M25

Figure S7: Asymmetric SNPs out of 5000 representative SNPs on chromosome 1, excluding A to G and C to T mutations.
